# Longitudinal changes in Super League match locomotor and event characteristics: A league-wide investigation over three seasons in rugby league

**DOI:** 10.1371/journal.pone.0260711

**Published:** 2021-12-02

**Authors:** Gordon Rennie, Brian Hart, Nicholas Dalton-Barron, Dan Weaving, Sean Williams, Ben Jones

**Affiliations:** 1 Carnegie Applied Rugby Research (CARR) Centre, Carnegie School of Sport, Leeds Beckett University, Leeds, United Kingdom; 2 Catapult Sports, Melbourne, Australia; 3 England Performance Unit, Rugby Football League, Leeds, United Kingdom; 4 Leeds Rhinos Rugby League Club, Leeds, United Kingdom; 5 Department for Health, University of Bath, Bath, United Kingdom; 6 School of Science and Technology, University of New England, Armidale, New South Wales, Australia; 7 Division of Exercise Science and Sports Medicine, Department of Human Biology, Faculty of Health Sciences, the University of Cape Town and the Sports Science Institute of South Africa, Cape Town, South Africa; Nottingham Trent University, UNITED KINGDOM

## Abstract

The 2019 and 2020 Super League (SL) seasons included several competition rule changes. This study aimed to quantify the difference between the 2018, 2019 and 2020 SL seasons for duration, locomotor and event characteristics of matches. Microtechnology and match event data were analysed from 11 SL teams, comprising 124 players, from 416 competitive matches across a three-year data collection period. Due to an enforced suspension of league competition as a consequence of COVID-19 restrictions, and subsequent rule changes upon return to play, season 2020 was divided into season 2020^a^ (i.e. Pre-COVID suspension) and season 2020^b^ (i.e. Post-COVID suspension). Duration, locomotor variables, and match events were analysed per whole-match and ball-in-play (BIP) periods with differences between seasons determined using mixed-effects models. There were significant (*ρ* ≤ 0.05) reductions in whole-match and BIP durations for adjustables and backs in 2019 when compared to 2018; albeit the magnitude of reduction was less during BIP analyses. Despite reduced duration, adjustables reported an increased average speed suggesting reduced recovery time between bouts. Both forwards and adjustables also experienced an increase in missed tackles between 2018 and 2019 seasons. When comparing 2019 to 2020^a^, adjustables and backs increased their average speed and distance whilst all positional groups increased average acceleration both for whole-match and BIP analyses. When comparing 2020^a^ to 2020^b^, all positional groups experienced reduced average speed and average acceleration for both whole-match and BIP analyses. Forwards experienced an increased number of tackles and carries, adjustables experienced an increased number of carries, and backs experienced an increased number of missed tackles when comparing these variables between season 2020^a^ and 2020^b^. Rule changes have a greater effect on whole-match duration and locomotor characteristics than those reported during BIP periods which suggests the implemented rule changes have removed stagnant time from matches. Amendments to tackle related rules within matches (e.g., introduction of the ‘six-again’ rule) increases the number of collision related events such as carries and tackles.

## Introduction

Since the mid-1950s, economists have stressed the importance of the uncertainty-of-outcome in sport and its role in maintaining spectator interest [1–3]. Changes to competition rules are one method of achieving this uncertainty amongst spectators [4] with the objective of ensuring that competitiveness still exists [5]. The Super League (SL) is the highest level of domestic rugby league competition in the northern hemisphere. Continued innovation is at the core its of strategy to raise the profile and appeal of the sport and to attract new audiences.

An example of this innovation within SL rugby league was the implementation of rule changes prior to the 2019 season which included reducing the number of player interchanges, introduction of the use of a shot-clock (for scrum, dropout or kick-at-goal) and an additional 10 minutes of playing time (referred to as ‘Golden Point’) in the event of a tied score upon full-time. Implementation of these rules aligned the SL rugby league competition with the Australasian National Rugby League (NRL) which is the premier rugby league competition within the southern hemisphere. Rule changes are not uncommon in sport [6]; however, their effects are rarely researched [7]. Modifications to rules are implemented with the intention of developing increased attractiveness of sport [8], reducing risk of injury [9], or match events (i.e. goals scored) [7]. The rule changes prior to the 2019 SL season were implemented with the intention of facilitating *“faster*, *more intense and more dramatic”* matches [10] thus demonstrating the competition’s alignment with the hypothesis of the uncertainty-of-outcome. Further rule changes were implemented moving from the 2019 to 2020 season which included a reduction in shot-clock accompanied by the assertion that *“the pace of rugby league will increase even further in 2020”* [11].

The majority of research within rugby league to date has been carried out within the NRL [12], although the largest study into match locomotor characteristics was recently undertaken in SL competition which included >7000 match observations with data analysed from ‘Project SL-Catapult’ [13]. Project SL-Catapult is a league-wide collaboration whereby all SL clubs are provided with the same Global Navigation Satellite System (GNSS) wearable tracking devices and proprietary software which allows for consistent data collection and league-wide analysis. These authors quantified the overall variability between matches, and found no overall effect between seasons, although positional differences were not explored [13]. A limitation of this study was that all variables analysed were displacement (i.e. locomotor) in nature thus did not include analysis of match events (e.g., tackles, missed tackles, and [ball] carries).

The lack of match events being reported alongside locomotor variables is common within rugby league [14–16]. This is problematic as analysis of locomotor variables alone presents a one-dimensional view of the sport that also involves collision based activity [17]. Previous longitudinal analyses of match events within rugby league competition have provided insight into evolutionary changes of both offensive and defensive involvements. When analysing offensive skill involvements from 2004 to 2014, there was a reduction (*large*) in the average number of offloads and play-the-balls and an increase (*large*) in the number of passes during competitive NRL matches [15]. Reporting both match events and locomotor variables together should provide practitioners with a more holistic evaluation of rugby league match characteristics.

Since the commencement of Project SL-Catapult in 2017, the sport of rugby league within the northern hemisphere has been subjected to numerous changes to competition rules from season-to-season. In addition to the goal of increasing the speed of matches [10, 11], rule changes have been introduced within rugby league competition with the objective of reducing potential SARS-CoV-2 transmission during the global COVID-19 pandemic [18]. In response to COVID-19 restrictions, the 2020 season was suspended for 141 days (15^th^ March 2020 to 2^nd^ August 2020) and upon return saw further rule changes introduced including the removal of scrums (a temporary rule change to reduce the risk of SARS-CoV-2 transmission) [18, 19] and the introduction of the ‘six again’ rule.

Given the rule changes in SL matches, understanding the recent trends would aid participating teams by ensuring physical preparation strategies are reflective of modern match demands and also allows the sport governing bodies to review the impact of the rule changes. Therefore, the aim of this study is to quantify the differences in duration, locomotor characteristics and match events between the 2018, 2019 and 2020 seasons.

## Methods

### Design

A prospective observational cohort design was used to compare rugby league match characteristics between the 2018, 2019 and 2020 SL seasons per positional group. Due to the enforced suspension of league competition as a consequence of COVID-19 restrictions, and subsequent rule changes upon return to play, season 2020 was divided into season 2020^a^ (i.e. Pre-COVID suspension) and season 2020^b^ (i.e. Post-COVID suspension). In addition to the rule changes implemented upon return to competition, further rationale for this separation of season 2020 is the length of break itself (e.g., the break between the end of season 2017 and beginning of season 2018 was 118 days, the break between the end of season 2018 and beginning of season 2019 was 111 days, the break between the end of season 2019 and beginning of season 2020 was 111 days; and the break due to COVID-19 enforced suspension was 141 days). Each season and sub-season had different rules during match play therefore the independent variable within this study were the four competitive seasons. The dependent variables were duration and locomotor characteristics (e.g., distance, average speed, high-speed [>5.5 m/s] running (HSR) distance, HSR distance per minute, and average acceleration), expressed over the whole-match and ball-in-play (BIP) periods, and the number of match events (e.g., tackles, missed tackles and carries). Variables were chosen due to their widespread use amongst practitioners working in within rugby league and their common usage in research studies [12, 20, 21].

### Participants

In line with previous research methodology, only players appearing in all seasons and sub-seasons were included in the study [17]. This resulted in a dataset of 124 unique male professional rugby league players that contained 4787 observations (season 2018 *n* = 1775, season 2019 *n* = 1812, season 2020^a^ *n* = 485 and season 2020^b^ *n* = 715). Participants were stratified according to their primary playing position for each match (i.e. the longest duration spent playing in that position during each individual match) and categorised into positional groups of forwards [props, hookers, second rows, and back rows] (*n* = 79, observations *n* = 2683; season 2018 *n* = 979, 2019 *n* = 1044, 2020^a^ *n* = 287, and 2020^b^ *n* = 373), adjustables [halves, fullbacks] (*n* = 36, observations *n* = 868; season 2018 *n* = 316, 2019 *n* = 322, 2020^a^ *n* = 84, and 2020^b^ *n* = 146), and backs [centres, outside backs] (*n* = 47, observations *n* = 1236; season 2018 *n* = 480, 2019 *n* = 446, 2020^a^ *n* = 114, and 2020^b^ *n* = 196). Anonymised data were analysed by the research team, and only summary statistics are presented, thus written informed consent was not needed by each participant. Informed consent was provided by the National Governing body. Ethics approval for the study was granted by Leeds Beckett University Ethics Committee.

### Data collection

Each player was fitted with the same GNSS microtechnology device (OptimEye S5, Catapult Sports, Melbourne, Australia) to derive the locomotor variables. The GNSS, sampling at 10 Hertz (Hz), provides geospatial positioning with global coverage encompassing both Global Positioning System (GPS) and Global Navigation Satellite System (GLONASS) constellations [22]. The test-retest reliability of OptimEye S5 microtechnology devices to measure instantaneous speed across a range of starting velocities has been reported to be acceptable (coefficient of variation [CV] = 2.0 to 5.3%) [23].

GNSS microtechnology data were initially downloaded and processed using proprietary OpenField^TM^ software (Catapult Sports, Melbourne, Australia) by a trained practitioner at each individual club. Subsequent querying of the OpenField^TM^ cloud database through an application program interface (API) provided access to data in the form of 10 Hz instantaneous speed and acceleration sampling points. These data points were processed for calculation of locomotor variables [13] which included distance, average speed (i.e. meterage per minute), HSR distance, HSR distance per minute and average acceleration.

Match events were coded by a commercial match statistics provider (Opta, Leeds, United Kingdom). Match events were extracted online (https://www.superleague.co.uk/match-centre) and included tackles, missed tackles and ball carries.

### Data filtering

The data pre-processing steps used by Dalton-Barron et al. [13] to derive locomotor variables were applied to the dataset which included filtering of the 10 Hz microtechnology data according to previously defined criteria (i.e. number of connected satellites ≥ 10, horizontal dilution of precision (HDOP) ≤ 2, velocity < 10 m∙s^-1^, acceleration < ±6 m∙s^-2^) [22, 24, 25]. Observations were excluded in instances where signal quality failed to meet these criteria during the data pre-processing phase (i.e. more than 10% of the raw file was filtered; observations = 1816). The mean number of connected satellites and mean HDOP from the finalised dataset was 11.5 ± 0.4 and 0.8 ± 0.2, respectively.

Following calculation of these locomotor variables, a synchronisation-point of the precise moment of kick-off was determined through visual inspection of latitude and longitude of each match alongside video footage. This allowed time synchronisation between the GNSS microtechnology data, match event data and allowed identification of contextual periods (i.e. match halves and BIP).

### Statistical analysis

To compare the magnitude of difference between playing seasons for each of the locomotor and match event variables, general linear mixed-effects (locomotor characteristics) were conducted using the *lmerTest* package in R [26] and generalised linear mixed effects models (match events) were conducted using the *lme4* package in R [27].

These were used as the variables within this study were nested in clusters of players, and crossed with fixtures, across four independent time periods. Fixed effects were included for season (i.e. 2018, 2019, 2020^a^ or 2020^b^) and positional category (forwards, adjustables, or backs), whilst the random effects were player and fixture. An alpha level of *ρ* ≤ 0.05 was set as the level of significance for all statistical tests.

Prior to analysis, inspection of normality was conducted per positional category through kernel density and quantile-quantile plots. For both whole-match and BIP analyses the distribution of several dependant variables was not normal; therefore, the median and quartile ranges (lower quartile [25%] and upper quartile [75%]) are presented. To reduce error arising from non-uniform residuals, locomotor variables were log-transformed prior to analysis, and subsequently back-transformed post-analysis [28].

For the linear mixed-effects models analysing locomotor characteristics, the magnitude and direction of difference between seasons (effect sizes [ES] ± 90% confidence limits [CLs]) were determined per playing position. The observed standard deviations (SDs) (pooled within- and between-player SDs) were multiplied by 0.2, 0.6 and 1.2 to anchor thresholds of *small*, *moderate* and *large* [29]. Effect size differences less than 0.2 were considered *trivial*. For the generalised linear mixed-effects models analysing event characteristics, rate ratios [RR] ± 90% CLs were used to compare between seasons for each positional group, with the distribution and link function contingent upon the nature of the dependent variable. The RR was classified as *trivial* (0.90 to 1.11), *small* (0.70 to 0.90 or 1.11 to 1.43), *moderate* (0.50 to 0.70 or 1.43 to 2.00) and *large* (<0.50 or >2.00) [30]. All statistical analyses were performed using R statistical software (R. 3.6.3, R Foundation for Statistical Computing, Vienna, Austria).

## Results

**Fig 1:**
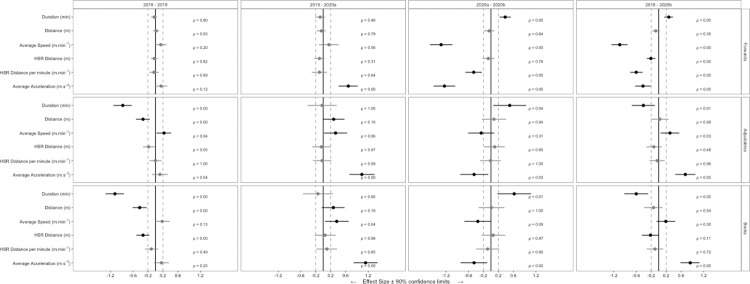
Standardised effect size (±90% confidence interval) differences in whole-match duration and locomotor characteristics between SL seasons 2018 to 2020^b^.

**Fig 2:**
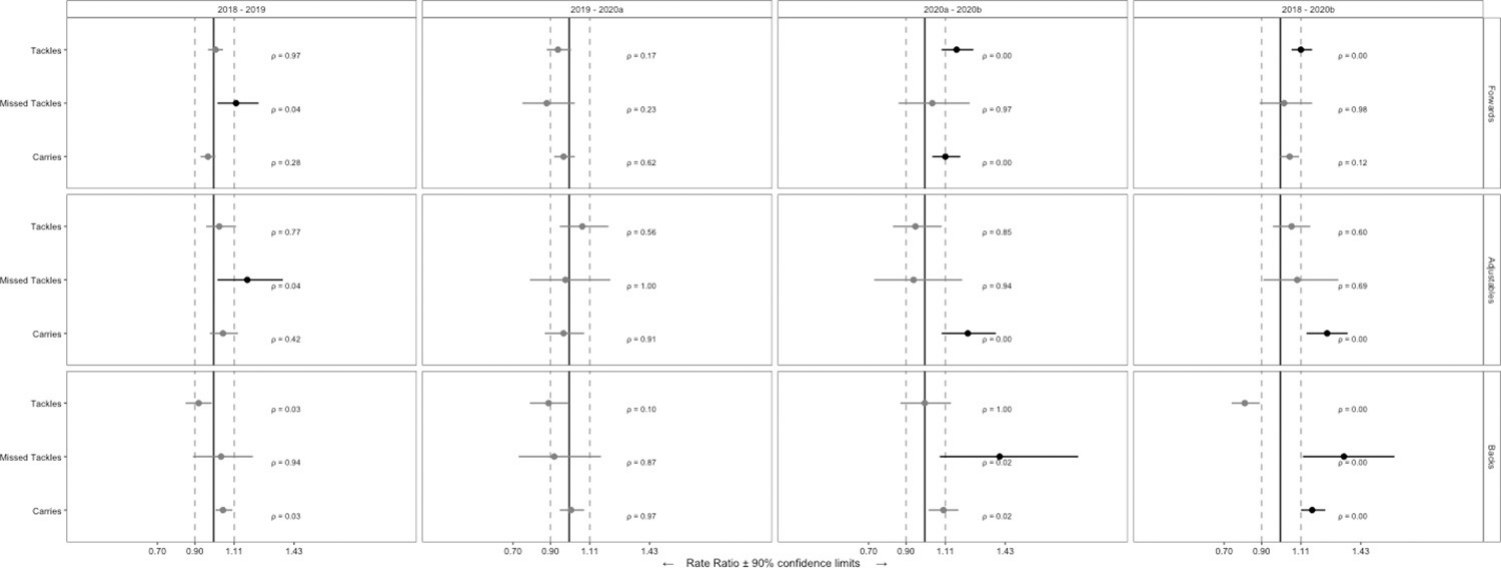
Standardised rate ratio (±90% confidence interval) differences in match events between SL seasons 2018 to 2020^b^.

**Fig 3:**
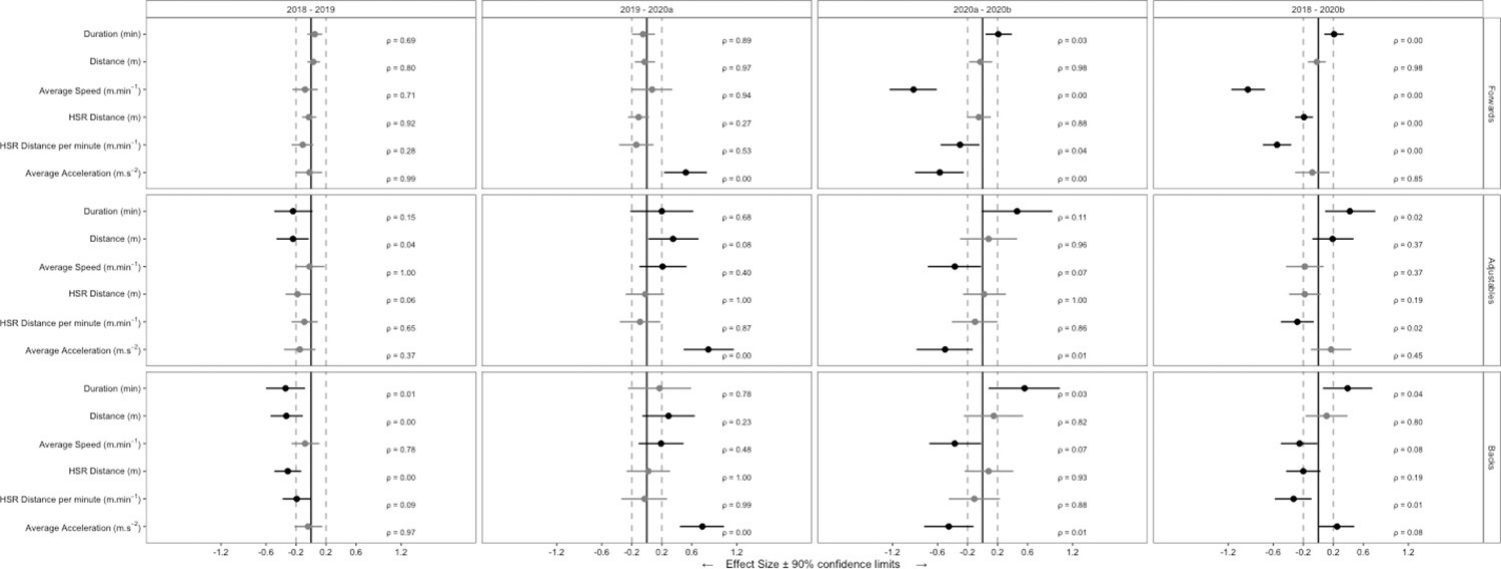
Standardised effect size (±90% confidence interval) differences in BIP duration and locomotor characteristics between SL seasons 2018 to 2020^b^.

A total of 4787 observations from 124 players were included in analysis. Descriptive whole-match duration, locomotor characteristics and match event data for seasons 2018, 2019, 2020^a^ and 2020^b^ are shown in Table 1. Descriptive BIP locomotor characteristics and match event data for seasons 2018, 2019, 2020^a^ and 2020^b^ are shown in Table 2. Fig 1 shows ES (± 90% CI) differences between seasons for whole-match duration and locomotor characteristics. Fig 2 shows RR (± 90% CI) differences between seasons for match events. Fig 3 shows ES (± 90% CI) differences between seasons for BIP duration and locomotor characteristics.

**Table 1:** Whole-match locomotor characteristics and match events stratified per positional category between seasons (Median [interquartile range]).

Position	Season	Duration (min)	Distance (m)	Average Speed (m/min)	HSR Distance (m)	HSR Distance per minute (m/min)	Average Acceleration (m/s/s)	Tackles	Missed Tackles	Carries
Forwards	2018	59.3 (43.3–80.4)	4458 (3335–6124)	77.2 (72.3–82.4)	238.0 (169.2–356.3)	4.42 (3.31–5.66)	0.39 (0.36–0.42)	26 (20–34)	2 (1–3)	10 (7–13)
	2019	58.3 (43.8–85.0)	4547 (3406–6434)	79.3 (74.1–85.2)	248.0 (168.2–378.0)	4.51 (3.40–5.67)	0.40 (0.37–0.43)	27 (21–34)	2 (1–3)	10 (7–13)
	2020^a^	57.6 (39.7–85.5)	4596 (3189–6668)	80.7 (75.9–85.7)	238.0 (156.2–359.4)	4.41 (3.18–5.59)	0.44 (0.41–0.46)	26 (19–32)	2 (1–3)	10 (7–13)
	2020^b^	82.5 (51.9–91.0)	5092 (3177–7077)	72.6 (58.8–79.4)	235.4 (129.2–408.9)	3.57 (2.41–5.07)	0.39 (0.32–0.42)	31 (25–38)	2 (1–3)	11 (8–14)
Adjustables	2018	95.8 (91.6–99.0)	7592 (7119–8016)	79.5 (74.9–84.2)	557.6 (440.8–682.1)	5.89 (4.84–7.37)	0.39 (0.36–0.42)	13 (8–18)	2 (1–3)	10 (7–13)
	2019	89.3 (86.3–92.8)	7414 (6919–7825)	81.6 (77.2–86.7)	556.4 (440.5–679.7)	6.24 (4.81–7.49)	0.39 (0.36–0.42)	13 (8–18)	2 (1–4)	11 (8–14)
	2020^a^	89.2 (87.2–91.2)	7646 (7279–7997)	85.9 (79.4–89.7)	526.3 (422.9–657.4)	5.94 (4.60–7.58)	0.44 (0.42–0.46)	14 (9–20)	2 (1–3)	10 (7–13)
	2020^b^	91.4 (89.6–95.2)	7566 (7194–7990)	81.2 (76.9–86.0)	527.7 (433.7–659.4)	5.75 (4.69–7.16)	0.42 (0.39–0.45)	14 (7–19)	2 (1–3)	12 (9–16)
Backs	2018	95.9 (92.1–99.0)	6961 (6509–7328)	72.8 (68.0–77.2)	606.9 (500.8–716.6)	6.43 (5.28–7.45)	0.36 (0.33–0.39)	8 (4–15)	1 (0–2)	13 (10–15)
	2019	89.6 (86.8–94.1)	6645 (6071–7040)	73.4 (67.2–78.0)	541.4 (452.3–644.3)	6.05 (4.94–7.05)	0.37 (0.33–0.39)	7 (3–13)	1 (0–2)	13 (11–16)
	2020^a^	89.1 (86.9–90.9)	6826 (6508–7138)	76.9 (71.5–80.3)	563.2 (445.6–681.6)	6.28 (4.88–7.41)	0.41 (0.39–0.43)	6 (3–11)	1 (1–2)	14 (11–16)
	2020^b^	91.9 (89.7–97.6)	6799 (6378–7210)	72.0 (67.8–77.2)	549.8 (462.8–647.9)	5.86 (5.01–7.04)	0.39 (0.37–0.41)	6 (3–11)	2 (1–3)	15 (12–17)

**Table 2:** Ball-in-play physical characteristics stratified per positional category between seasons (Median [interquartile range]).

Position	Season	Duration (min)	Distance (m)	Average Speed (m/min)	HSR Distance (m)	HSR Distance per minute (m/min)	Average Acceleration (m/s/s)
Forwards	2018	35.2 (26.2–49.2)	3520 (2663–4869)	101.2 (95.0–107.9)	228.2 (160.1–337.7)	6.95 (5.15–9.05)	0.57 (0.52–0.61)
	2019	35.6 (27.2–50.4)	3647 (2730–5060)	101.7 (94.7–108.8)	236.8 (160.4–357.8)	6.97 (5.15–8.83)	0.57 (0.52–0.61)
	2020a	35.6 (24.9–54.1)	3738 (2533–5316)	102.4 (95.8–108.8)	226.5 (151.2–345.2)	6.66 (4.95–8.39)	0.60 (0.57–0.63)
	2020^b^	46.3 (29.8–57.6)	4032 (2470–5704)	94.4 (84.3–101.0)	225.8 (122.6–393.4)	5.58 (4.00–7.81)	0.56 (0.52–0.60)
Adjustables	2018	57.7 (54.1–61.3)	5860 (5425–6225)	102.2 (95.8–106.9)	517.8 (401.7–641.1)	9.05 (7.18–11.2)	0.55 (0.51–0.58)
	2019	55.7 (51.2–59.7)	5686 (5227–6136)	102.1 (96.0–108.5)	515.9 (397.2–625.4)	9.31 (7.10–11.4)	0.54 (0.49–0.57)
	2020^a^	56.8 (54.8–58.3)	5966 (5440–6325)	104.7 (99.5–109.3)	491.5 (385.8–620.7)	8.50 (6.72–10.9)	0.58 (0.54–0.60)
	2020^b^	60.2 (56.9–64.7)	5931 (5579–6283)	98.3 (94.1–104.8)	490.1 (414.5–587.3)	8.32 (6.77–9.91)	0.56 (0.52–0.59)
Backs	2018	57.8 (54.9–61.5)	5230 (4800–5596)	90.2 (84.5–95.7)	553.2 (441.8–656.8)	9.60 (7.78–11.3)	0.49 (0.44–0.53)
	2019	56.3 (51.4–60.4)	4971 (4494–5402)	89.0 (81.8–94.9)	499.8 (406.9–595.7)	8.86 (7.23–10.8)	0.48 (0.44–0.52)
	2020^a^	56.8 (54.2–59.3)	5177 (4780–5527)	90.1 (84.9–97.0)	511.8 (404.6–597.5)	8.73 (7.16–10.7)	0.52 (0.49–0.56)
	2020^b^	60.3 (57.4–64.3)	5272 (4893–5648)	86.7 (81.9–91.2)	512.2 (412.6–603.6)	8.44 (6.87–10.0)	0.49 (0.46–0.54)

### Changes in whole-match and ball-in-play duration

For forwards, whole-match and BIP duration increased (*small*) between 2020^a^ and 2020^b^. For adjustables, whole-match duration reduced (*moderate*) between 2018 and 2019 but increased (*small*) between 2020^a^ and 2020^b^. BIP duration reduced (*small*) between season 2018 and 2019 however increased (*small*) from 2019 to 2020^a^ and 2020^a^ to 2020^b^ for adjustables. For backs, whole-match duration reduced (*moderate*) between 2018 and 2019 but increased (*moderate*) between 2020^a^ and 2020^b^. BIP duration reduced (*moderate*) for backs between 2018 and 2019 however increased (*moderate*) between 2020^a^ to 2020^b^.

Between 2018 to 2020^b^; for forwards, whole-match and BIP duration increased (*small*), and whole-match duration reduced (*small*) but BIP duration increased (*small*) for adjustables. For backs, whole-match duration reduced (*moderate*) however BIP duration increased (*small*). All other whole-match and BIP comparisons were *trivial*.

### Changes in whole-match and ball in-play locomotor variables

For forwards, whole-match average speed reduced (*large*) and BIP average speed reduced (*moderate*) between 2020^a^ and 2020^b^. For adjustables, whole-match distance reduced (*small*) and average speed increased (*small*) between 2018 and 2019; however, BIP distance was the only dependent variable to reduce (*small*) in this same period. From 2019 to 2020^a^, whole-match and BIP distance and average speed increased (*small*) for adjustables. Whole-match and BIP average speed reduced (*small*) between 2020^a^ and 2020^b^ for adjustables. For backs, whole-match and BIP distance reduced (*small*) between 2018 and 2019. Between 2019 and 2020^a^, for whole-match and BIP periods, total distance and average speed increased (*small*). When comparing 2020^a^ and 2020^b^ for backs, whole-match and BIP periods, average speed reduced (*small*).

High-speed running distance per minute reduced (*small*) for forwards both for whole-match and BIP analysis 2020^a^ and 2020^b^; however, all other whole-match and BIP comparisons were *trivial*.

For forwards, whole-match average acceleration reduced (*large*) and BIP average acceleration reduced (*small*) between 2020^a^ and 2020^b^. For adjustables, whole-match and BIP average acceleration increased (*moderate*) from 2019 and 2020^a^. However, average acceleration reduced (*moderate*) for adjustables for both whole-match and BIP periods between 2020^a^ and 2020^b^. For backs, whole-match and BIP average acceleration increased (*moderate*) between 2019 and 2020^a^; however whole-match and BIP average acceleration reduced (*small*) for 2020^a^ and 2020^b^ comparison. All other whole-match and BIP comparisons were *trivial*.

When comparing data from season 2018 to season 2020^b^; for forwards, whole-match average speed reduced (*moderate*), HSR distance reduced (*small*), HSR distance per minute reduced (*moderate*) and average acceleration reduced (*small*). Forwards BIP average speed reduced (*moderate*) and BIP HSR distance per minute reduced (*small*). For adjustables, whole-match average speed increased (*small*) and average acceleration increased (*moderate*) whilst BIP HSR distance per minute increased (*small*). For backs, whole-match HSR distance reduced (*small*) and average acceleration increased (*moderate*). Backs BIP average speed reduced (*small*), BIP HSR distance reduced (*small*), BIP HSR distance per minute reduced (*small*) and BIP average acceleration increased (*small*).

### Changes in match events

For forwards, the number of missed tackles increased (*small*) from 2018 to 2019 and the number of tackles and carries increased (*small*) from season 2020^a^ to 2020^b^. For adjustables, the number of missed tackles increased (*small*) from 2018 to 2019 and the number of carries increased (*small*) from 2020^a^ to 2020^b^. For backs, the number of missed tackles increased (*small*) from 2020^a^ to 2020^b^. All other match events comparisons were *trivial*.

Between 2018 to 2020^b^, for forwards, the number of tackles increased (*small*). For adjustables, the number of carries increased (*small*). For backs, the number of missed tackles increased (*small*) as did the number of carries (*small*).

## Discussion

Given the implementation of several rule changes within SL rugby league matches in 2019 (i.e. reduced number of interchanges from 10 to 8, introduction of the shot-clock, and golden point), 2020^a^ (i.e. reduction in the shot-clock by five seconds; 30 seconds for a scrum and 25 seconds for a drop-out), and 2020^b^ (i.e. temporary removal of scrums and introduction the ‘six again’ rule), the aim of this study was to quantify differences between 2018 to 2020^b^ seasons for player locomotor characteristics and match events.

This is the first study to include league-wide data for locomotor characteristics and match events across multiple league seasons (i.e. three-year period). The study found that rule changes have a greater effect on whole-match characteristics than those reported when the ball is in play, suggesting the rule changes have removed stagnant time from matches. Findings suggest that, from 2018 to 2020^b^, the SL have achieved their objective in increasing the speed of matches as supported by increased average speed and average acceleration.

### Season 2018–2019

Findings from this league-wide study suggest that the 2019 SL rule changes had a more apparent effect on whole-match characteristics than BIP periods. Adjustables and backs experienced a reduction in whole-match duration in season 2019 compared to 2018; however, when data were analysed for BIP periods the reduction in duration was of a lower magnitude. Forwards did not experience any change in duration which is likely due to the shot-clock having a greater impact upon positional groups that have greater match durations (e.g., forwards are more commonly interchanged [31, 32]). Despite reduced match duration for adjustables from 2018 to 2019, this study found average speed increased. This is likely because the 2019 SL rule changes purposefully reduce the amount of inactive time or ‘stagnated’ play within rugby league competition [16], particularly with the introduction of the shot-clock. Players therefore likely have reduced time between bouts, which has implications for training practices and when preparing players for the match demands [33].

The present study demonstrated an increase in the number of missed tackles within the 2019 season compared to 2018 for forwards and adjustables. This may be due to the reduced rest time during matches (i.e. reduced [*moderate*] whole-match duration however only reduced [*small*] BIP duration) and reduced ability to interchange players which potentially contributes to more within-match fatigue. The observed increase in number of missed tackles is in agreement with previous research in the NRL [16]. Using multidimensional scaling, Woods et al. [16] found that within the NRL competition in the seasons following 2015, the number of missed tackles and tackle breaks increased suggesting that matches were becoming more ‘free flowing’. The NRL implemented the same shot-clock rule change as well as fewer permitted number of interchanges in 2016 to reduce ‘stagnated’ play. These authors suggested the increase in missed tackles was a possible consequence of this rule change, and specifically due to an increased number of offloads completed in attack.

### Season 2019 – 2020^a^

Ahead of season 2020^a^, with a view to further increasing the speed of match-play [11], the shot-clock was reduced by five seconds (i.e. 30 seconds for a scrum and 25 seconds for a drop-out were now allowed). Whole-match duration for all positions appears unaffected by this rule change; however, both adjustables and backs experienced an increase in distance and average speeds. Both these positional groups also experienced an increase in distances and average speeds for BIP periods. All positional groups experienced increases in whole-match and BIP for average acceleration.

Findings from this comparison suggest that the reduction of time from the shot-clock successfully contributed to further increasing the speed of SL matches. However, this should be interpreted with caution as this data collection period within the study contained the smallest number of observations across all seasons.

### Season 2020^a^ – 2020^b^

The most substantial difference in dependent variables was observed during this comparative period for forwards whereby average speed and average acceleration reduced both during whole-match and BIP analyses. During this comparative period, adjustables and backs also experienced a reduction in whole-match average speed and average acceleration. Once matches returned from temporary league suspension in August 2020 (i.e. season 2020^b^) they did so with two notable rule changes. The ‘six-again’ rule, which allows the referee to award a new set of six tackles instead of a penalty was introduced alongside the temporary removal of scrums. Scrums were removed with the objective of reducing the likelihood of SARS-CoV-2 transmission during matches [18]. Whole-match and BIP duration increased for all positional groups in 2020^b^ when compared to 2020^a^. This increase in duration coupled with no change in distances covered demonstrates that the average speed of matches reduced between season 2020^a^ and 2020^b^.

Although the average speed of matches appears to have reduced, an increase in the number of tackles and carries for forwards was observed. Furthermore, adjustables reported an increase in the number of carries and backs experienced an increase in the number of missed tackles which suggests greater collision involvements were experienced by all positional groups. Previous research has demonstrated reduced average running speed and reduced average acceleration as number of collisions increases [34, 35]. This increase in match events may be attributed to the ‘six-again’ rule which allows the referee to award six more tackles in the event of an infringement by a defending side. These findings are consistent with previous research which suggests that, due to an inability to cover large distances whilst being involved in tackles or carries, locomotor characteristics are likely to reduce as collision frequency increases [36].

### Season 2018 – 2020^b^

Findings when comparing characteristics and events from season 2018 to those experienced in 2020^b^ demonstrate that whole-match and BIP HSR distance and HSR distance per minute has reduced for forwards whilst HSR distance per minute has reduced for adjustables and backs. Season 2018 to 2020^b^ comparison is not the first-time backs have been shown to reduce high velocity activity within rugby league. The effects of a rule amendment known as the ‘10-meter’ rule change within rugby league has been previously investigated with results suggesting that sprinting activities marginally increased amongst forwards (0.5%) and reduced within backs (-0.8%) [6]. However, Meir et al. [6] did not provide a magnitude of change therefore the effects of this rule change remain unknown. Previous research has shown that HSR distances can be impacted upon by altering pitch dimensions [37]. Pitch dimensions and number of players on the field at any given time remained consistent between SL seasons which may provide an explanation for this variable less affected during the observational period in this study.

Forwards have shown a reduction in average speed and average acceleration when comparing 2020^b^ characteristics to those within season 2018; however, have also completed more tackles within 2020^b^ when compared to 2018. The reduction in average speed and average acceleration suggests that players are downregulating their physical output, given that the number of tackles undertaken have increased. Adjustables and backs have experienced a reduction in whole-match duration however both positional groups have experienced an increase in carries. Backs have also reported a reduction in tackles and an increase in missed tackles. This general increase in match events may be explained by the increase in BIP duration under the logic that increased BIP duration provides more opportunity for events such as tackles, carries and missed tackles to occur. The notable exception to this observation is within backs which experienced a reduced number of tackles in season 2020^b^ when compared to 2018.

Whilst direct comparison with previous research is challenging due to methodological differences, this does appear to contradict general findings from previous literature using observations from the NRL. Gabbett and Hulin [15] reported that the number of tackles increased by 5% (ES = 0.94) from 2004 (297 ± 16) to 2014 (313 ± 12), suggesting that NRL matches gradually became more defence orientated. From seasons 2012 to 2014, Evans et al. [17] reported an increased number of collisions within a newly promoted SL franchise however their study was not without limitation (e.g., data was collected from a single team containing a relatively small sample size).

A limitation within the present study is that data derived from wearable technology is only available from season 2018 onwards and therefore typical season-to-season variability, independent of rule changes is unknown. It is therefore unclear whether the change between seasons is outside of this variability.

The exclusion of data within this study based on ‘signal quality’ (i.e. number of satellites and HDOP) is in keeping with accepted methodologies used in previous sports science literature. However, it’s important to acknowledge from an engineering perspective that a good HDOP value does not guarantee more accurate positional data since HDOP measures only one source of error (i.e. the satellite configuration). A more common issue within an urban and indeed elite sport environment is that of multipathing which occurs when satellite signals are reflected from building or stadium surfaces (i.e. grandstands) [38, 39]. HDOP is calculated purely from the geometrical organisation of the satellites [22] therefore multipathing does not change the HDOP values reported. Future research within sports science should look to better understand the consequences that multipathing may have upon the quality of wearable derived GNSS data.

Whilst the wearable technology used within this study to derive human locomotion has previously been shown to be valid and reliable, a limitation is the unknown inter- and intra-reliability of Opta personnel when coding rugby league match events. Whilst inter-operator reliability of event coding has been demonstrated to be very good within soccer [40] and AFL [41], it is unclear if these findings transcend other sports and therefore future research should aim to determine this.

Finally, rugby league appears to review and modify rules on an annual basis, therefore whilst specific findings may appear outdated for practitioners once new rules are implemented, evaluating the trends following rule changes helps policymakers understand the potential impact of new rules in the future (e.g., if further changes are made to the number of interchanges).

## Conclusion

The aim of this study was to quantify differences in duration, locomotor characteristics and match events between 2018, 2019, 2020^a^ and 2020^b^ seasons following regular rule changes within rugby league competition. The study found that rule changes have a greater effect on whole-match characteristics than those recorded during BIP periods, suggesting the rule changes have removed stagnant time from matches. Moving sequentially from seasons 2018 through to 2020^b^, the SL appear to have achieved their objective in increasing the speed of matches as shown by increased average speeds and average acceleration. Amendments to tackle related rules within matches (e.g., introduction of the ‘six-again’ rule) increase the number of collision related events such as carries and tackles which players are exposed to. Further research should aim to determine if there is a cause-and-effect relationship exists between fatigue accumulated with increased BIP duration and reduced whole-match average speed.
